# Mapping of fatty acid composition with free‐breathing MR spectroscopic imaging and compressed sensing

**DOI:** 10.1002/nbm.4241

**Published:** 2020-01-03

**Authors:** James A. Rioux, Miriam Hewlett, Christa Davis, Chris V. Bowen, Kimberly Brewer, Sharon E. Clarke, Steven D. Beyea

**Affiliations:** ^1^ Biomedical Translational Imaging Centre Halifax Nova Scotia Canada; ^2^ Department of Diagnostic Radiology Dalhousie University Halifax Nova Scotia Canada; ^3^ Department of Physics and Atmospheric Science Dalhousie University Halifax Nova Scotia Canada; ^4^ Department of Medical Biophysics University of Western Ontario London Ontario Canada; ^5^ School of Biomedical Engineering Dalhousie University Halifax Nova Scotia Canada

**Keywords:** compressed sensing, fat composition, fatty liver disease, preclinical imaging, spectroscopic imaging

## Abstract

Nonalcoholic fatty liver disease (NAFLD) is a growing health problem, and a major challenge in NAFLD management is identifying which patients are at risk of progression to more serious disease. Simple measurements of liver fat content are not strong predictors of clinical outcome, but biomarkers related to fatty acid composition (ie, saturated vs. unsaturated fat) may be more effective. MR spectroscopic imaging (MRSI) methods allow spatially resolved, whole‐liver measurements of chemical composition but are traditionally limited by slow acquisition times. In this work we present an accelerated MRSI acquisition based on spin echo single point imaging (SE‐SPI), which, using appropriate sampling and compressed sensing reconstruction, allows free‐breathing acquisition in a mouse model of fatty liver disease. After validating the technique's performance in oil/water phantoms, we imaged mice that had received a normal diet or a methionine and choline deficient (MCD) diet, some of which also received supplemental injections of iron to mimic hepatic iron overload. SE‐SPI was more resistant to the line‐broadening effects of iron than single‐voxel spectroscopy measurements, and was consistently able to measure the amplitudes of low‐intensity spectral peaks that are important to characterizing fatty acid composition. In particular, in the mice receiving the MCD diet, SE‐SPI showed a significant decrease in a metric associated with unsaturated fat, which is consistent with the literature. This or other related metrics may therefore offer more a specific biomarker of liver health than fat content alone. This preclinical study is an important precursor to clinical testing of the proposed method.

MR‐based quantification of fatty acid composition may allow for improved characterization of non‐alcoholic fatty liver disease. A spectroscopic imaging method with appropriate sampling strategy allows whole‐liver mapping of fat composition metrics in a free‐breathing mouse model. Changes in metrics like the surrogate unsaturation index (UIs) are visible in mice receiving a diet which induces fat accumulation in the liver, as compared to a normal diet; such metrics may prove useful in future clinical studies of liver disease.

## Graphical abstract

MR‐based quantification of fatty acid composition may allow for improved characterization of non‐alcoholic fatty liver disease. A spectroscopic imaging method with appropriate sampling strategy allows whole‐liver mapping of fat composition metrics in a free‐breathing mouse model. Changes in metrics like the surrogate unsaturation index (UIs) are visible in mice receiving a diet which induces fat accumulation in the liver, as compared withto a normal diet; such metrics may prove useful in future clinical studies of liver disease.

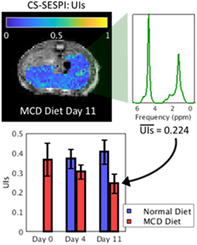

**Abbreviations used**
BW: bandwidth
CIRCUS: CIRcular Cartesian UnderSampling
CS: compressed sensing
FOV: field of view
HSVD: Hankel singular value decomposition
MCD: methionine and choline deficient
MRSI: magnetic resonance spectroscopic imaging
NAFLD: nonalcoholic fatty liver disease
NASH: nonalcoholic steatohepatitis
PDFF: proton density fat fraction
PRESS: point resolved spectroscopy
PUI: polyunsaturation index
ROI: region of interest
SE‐SPI: spin echo single point imaging
SPIO: superparamagnetic iron oxide
SVS: single‐voxel spectroscopy
TE: echo time
TR: repetition time
UI: unsaturation index
UIs: surrogate unsaturation index

## 1 INTRODUCTION

Nonalcoholic fatty liver disease (NAFLD) is on the rise globally; according to several estimates, 25%‐40% of the populations in developed countries have NAFLD to some extent ^1^, ^2^, ^3^. While most instances of NAFLD have a relatively benign course, in 10%‐20% of cases there is a significant risk of progression to more serious forms of disease, such as nonalcoholic steatohepatitis (NASH) and cirrhosis, as well as an increased risk of cancer ^2^, ^4^. Early identification of patients at high risk of these complications is essential for proper management of NAFLD, including the introduction of lifestyle changes or medications to slow progression ^3^.

The clinical gold standard for detection of liver disease and characterization of its severity is biopsy, but this carries risks due to its invasive nature, and is therefore not ideal for evaluating low‐risk patients or for following the progression of disease over time ^5^. Biopsies also sample tissue from a limited area and may mischaracterize heterogeneous liver disease; for example, paired biopsy samples in the same patient can disagree upon the stage of NASH in up to 40% of individuals ^6^. Ideally, a noninvasive whole‐liver imaging approach such as fat quantification with magnetic resonance imaging (MRI) would be used to assess the risk of more serious complications in patients known or suspected to have NAFLD.

One common approach, which is increasingly used in clinical settings, is to measure the proton density fat fraction (PDFF) using a chemical shift‐encoded imaging sequence ^7^. Studies using MRI to measure PDFF in NAFLD have shown high accuracy compared with pathology ^8^, and have demonstrated variability in the distribution of liver fat, which increases proportionately with the degree of fat accumulation ^9^, highlighting the need for a spatially resolved measurement. However, while PDFF is strongly correlated with steatosis ^8^, it has also been shown that PDFF alone is not a reliable predictor for whether a given individual with NAFLD will progress to NASH ^10^, ^11^. MR elastography has shown promise for detecting liver fibrosis ^12^, which is diagnostic of NASH if present in the setting of NAFLD, but MRE hardware is not available at all sites. Additional imaging biomarkers that can assist in distinguishing benign and progressive fatty liver disease are therefore desirable.

There is evidence that the composition of liver fat, ie, the proportions of saturated and unsaturated fatty acids, may indicate patients at risk of disease progression ^13^, ^14^. Specifically, it is hypothesized that a decrease in the relative concentration of polyunsaturated fatty acids in the liver contributes to the development of NASH by downregulating genes associated with the breakdown of fatty acids, and upregulating genes associated with their synthesis, contributing to steatosis and eventually steatohepatitis ^13^, ^15^. Measurements of liver fat in NAFLD patients with biopsy ^16^ or gas‐liquid chromatography ^17^ have shown a higher concentration of saturated fatty acids, and a corresponding decrease in polyunsaturated fatty acids, compared with healthy controls. However, these types of measurements are not suitable for routine screening.

MR spectroscopy offers opportunities to examine not only the concentration of fat in liver tissue, but also the relative fraction of different lipid types, by measurement of the various peaks in the fat spectrum ^13^, ^18^. Such measurements may therefore serve as noninvasive indicators of NAFLD severity or progression that are more clinically translatable. The disadvantage of single‐voxel spectroscopy (SVS) from a clinical standpoint is similar to biopsy: unless multiple voxels are obtained, it is possible that a region will be sampled which is not representative of the entire liver, or which misses areas of disease.

Spectroscopic imaging approaches ^19^, ^20^ acquire the complete fat spectrum at every location within an image, and can provide higher resolution (ie, smaller effective voxel sizes) than spectroscopy alone, but also incur a significant time penalty and are therefore not in widespread clinical use. However, acceleration techniques such as compressed sensing (CS) ^21^, ^22^, ^23^ have made it possible for spectroscopic images to be acquired in clinically viable scan times. This creates opportunities to use spectroscopic imaging as an adjunct scan to provide additional information on the specific composition of liver fat in those patients demonstrated to have high PDFF indicative of liver steatosis (ie, >6.4% ^8^).

In this work we demonstrate the ability of spin echo single point imaging (SE‐SPI) ^20^, ^24^ to produce maps of fatty acid composition in phantoms and in a mouse model of fatty liver disease. An appropriate pseudo‐random undersampling strategy and reconstruction with CS allows for a free‐breathing scan with retrospective respiratory compensation. This validation study will serve as an important precursor to eventual clinical deployment and the testing of such techniques.

## 2 METHODS

### 2.1 Free‐breathing SE‐SPI

Spectroscopic imaging approaches are distinguished from traditional imaging sequences by the lack of a readout gradient during data collection, allowing the complete signal evolution to be sampled at each location in the image. In the case of SE‐SPI, a 180‐degree refocusing pulse is applied to reverse signal decay due to magnetic field inhomogeneity, which is important in liver imaging due to the high iron concentrations that can accompany fatty liver disease ^25^. Because the SE‐SPI image is completely phase‐encoded, fast T_2_* relaxation due to iron overload will broaden the spectral lines but will not produce blurring or other artifacts in the corresponding images ^24^.

**FIGURE 1:**
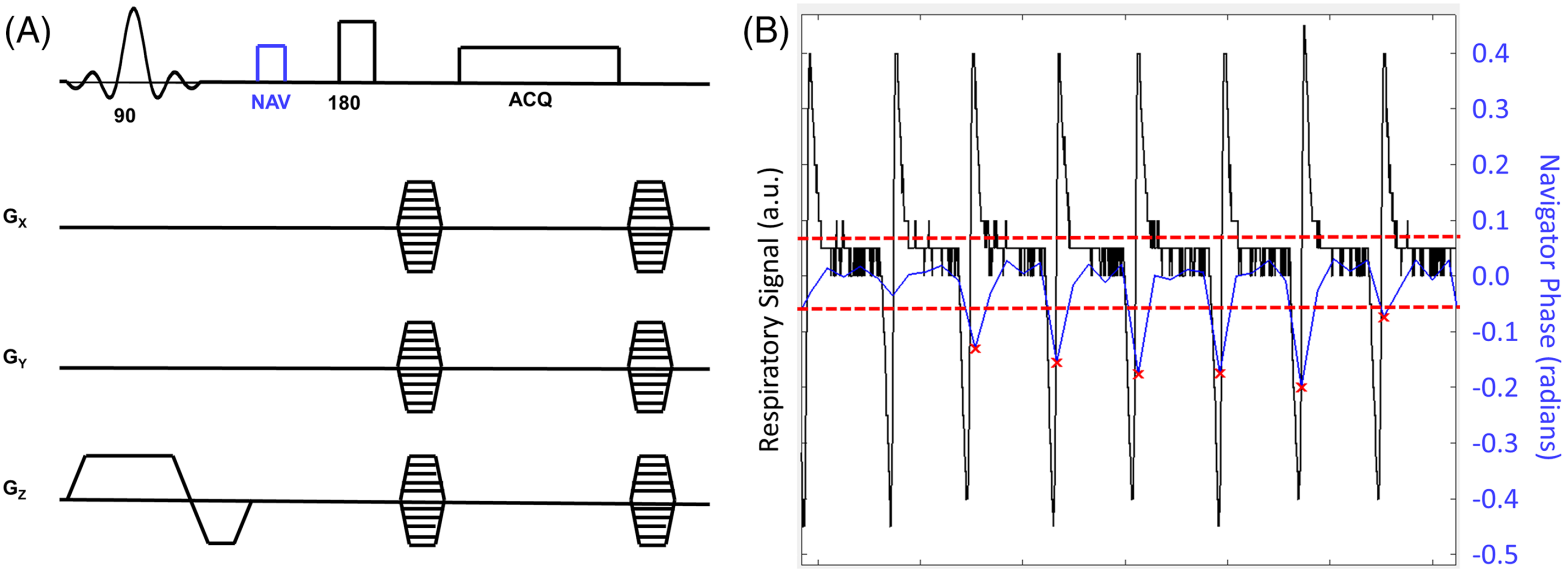
(A) Spin echo single point imaging (SE‐SPI) sequence diagram. Navigator signal is collected after excitation and slice select rewinder gradients, and before phase encoding. (B) Comparison of navigator signal (blue) with concurrently acquired respiratory signal in a mouse obtained from an abdominal bellows (black). Phase encodes whose navigator value is greater than one standard deviation from the mean are not used for reconstruction since they correspond to periods of respiratory motion; the acceptance range is indicated with red dashed lines while the red Xs denote phase encodes omitted from the reconstruction

Any sequence used for in vivo abdominal imaging must provide compensation for respiratory motion during the scan. In our SE‐SPI implementation, several navigator points are acquired at the start of each readout prior to phase encoding, and these are used to retrospectively remove samples corrupted by motion. A moving average of the navigator phase is computed, and any points that deviate by more than one standard deviation from that average are discarded. The pulse sequence is illustrated in Figure 1, along with a representative navigator signal over several respirations, demonstrating that discarded points correspond to periods of motion as measured by an abdominal bellows.

**FIGURE 2:**
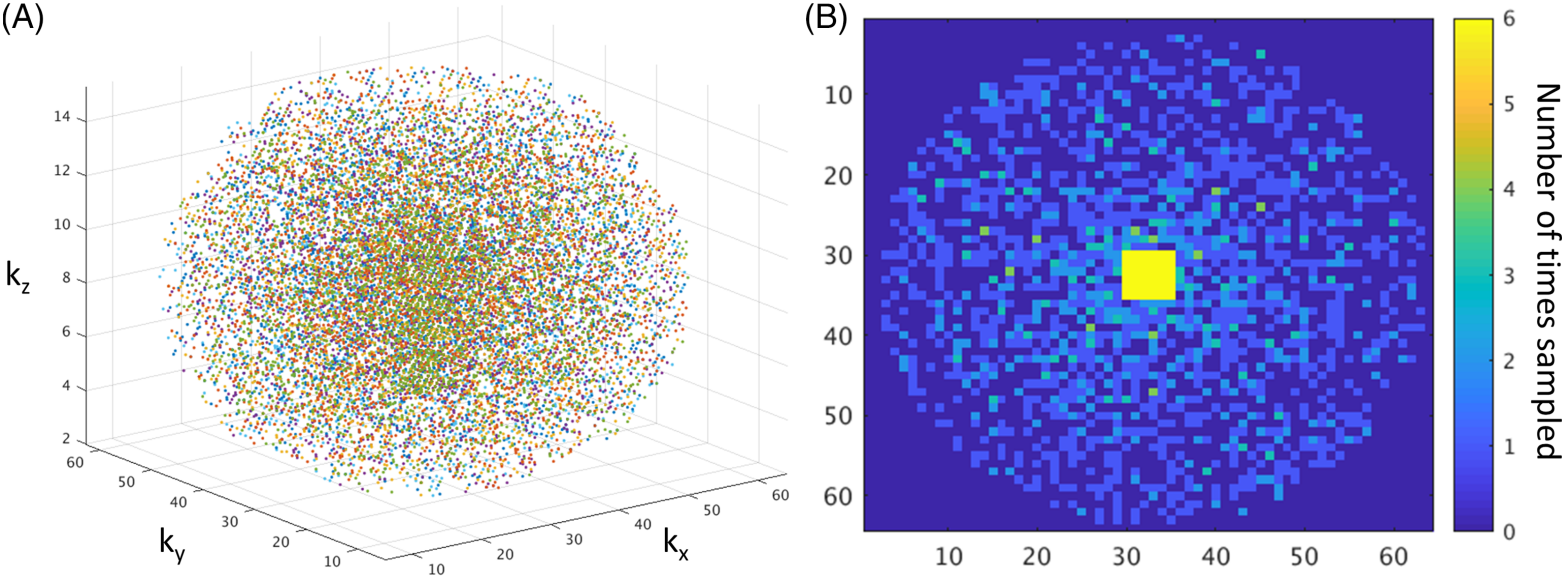
(A) Scatter plot of all sampled k‐space locations in a typical sampling 3D spin echo single point imaging (SE‐SPI) sampling prescription. In this example, six different overlapping patterns are acquired; all points within a pattern are unique and acquired in central order; points may be repeated in subsequent patterns. (B) Degree of sampling for k‐space locations in the central kx‐ky plane; points in the central 6 x 6 square are sampled in every pattern but most other locations are sampled 0‐2 times over the course of the acquisition

To ensure that the central regions of k‐space are fully sampled even after respiratory compensation, the center of k‐space is oversampled while the periphery is randomly undersampled using a pattern based on CIRCUS ^26^. CIRCUS (CIRcular Cartesian UnderSampling) is a golden‐angle sampling strategy and therefore ensures near‐optimal coverage of k‐space even after removal of an unknown number of samples during retrospective navigator correction. CIRCUS samples k‐space along nested squares on a Cartesian grid, meaning it does not require gridding or nonuniform Fourier reconstruction, and the sampling pattern is faster to compute than alternatives such as Poisson disc ^26^. Because SE‐SPI is purely phase‐encoded, there is an opportunity to undersample in all three spatial dimensions, but this requires an extension of the CIRCUS technique to 3D. In our implementation, points are selected from nested spherical shells; the nth shell contains all points in an N x N x N matrix within a distance

(1) $$R_{n}=\sqrt{\mathrm{nN}/2+9}$$

of the k‐space center, sorted by zenith and azimuthal angle. This formula has been empirically devised to yield a fairly constant number of points per shell on the periphery of k‐space such that those regions are not too highly undersampled, while also ensuring that the central region is fully sampled. Examples of sampling patterns are shown in Figure 2; generally, multiple nonidentical sampling patterns are acquired sequentially to oversample the k‐space center.

### 2.2 Spectral analysis

Analysis and reconstruction of spectra obtained with SE‐SPI, or with a point resolved spectroscopy (PRESS) acquisition for comparison, were performed in Matlab 2017b (Mathworks, Natick, MA). Characterization of fat spectra used the Hankel singular value decomposition (HSVD) ^27^, which identifies a specified number of components in the input signal and returns their frequency, phase, magnitude and dispersion. Tests on phantoms demonstrated that searching for 25 HSVD components provided the highest intrascan reliability (data not shown). Identification of components was not guided by prior knowledge of the peak frequencies, but, after the HSVD algorithm was completed, the identified components were assigned to the known peak locations ^13^, ^28^. If multiple components were assigned to a given peak, a complex sum of all components was taken, and then a Lorentzian function was fitted to the result in order to obtain an amplitude and phase for the composite peak ^29^.

For a given spectrum, HSVD analysis permits computation of the fat fraction and the relative amplitudes of spectral peaks. Based on these values it is possible to compute various metrics related to the degree of saturation of the fatty acid chains; these metrics are candidates for a biomarker to distinguish benign from potentially progressive disease. While several such metrics have been defined in the literature ^14^, ^26^, ^30^, ^31^, in this work we use the metrics proposed in ^13^, which compute the following indices:

(2A) $$\mathrm{UI}=A_{5.3}/\left(A_{5.3}+A_{1.8}+A_{1.3}+A_{0.9}\right)$$

(2B) $$\mathrm{UIs}=\left(A_{1.8}+A_{2.8}\right)/\left(A_{5.3}+A_{1.8}+A_{1.3}+A_{0.9}\right)$$

(2C) $$\mathrm{PUI}=A_{2.8}/\left(A_{1.8}+A_{2.8}+A_{1.3}+A_{0.9}\right)$$

where A_x_ is the relative amplitude of the peak at a chemical shift of approximately x ppm. These values represent the unsaturation index (UI, based on the methene peak), a surrogate unsaturation index (UIs, based on the allylic and diallylic methylene peaks around 2‐3 ppm), which is useful when the 5.3 ppm methene peak is unreliable, and the polyunsaturation index (PUI, based on the diallylic methylene peak) ^13^.

### 2.3 Image reconstruction

Undersampled data were reconstructed with Blind‐CS ^32^. In this formulation, the temporal evolution at each pixel **x** is expressed as a linear combination of R basis functions,

(3A) $$S\left(x,t\right)=\sum_{i=1}^{i=R}u_{i}\left(x\right)v_{i}\left(t\right)\;$$

The Casorati matrix of the entire M‐pixel dataset can then be expressed as the product of a matrix U containing all the coefficients (assumed sparse), and a dictionary V of basis functions of length N:

(3B) $$\left(\begin{array}{ccc} S\left(x_{1},t_{1}\right) & \cdots & S\left(x_{1},t_{N}\right)\\ \vdots & \ddots & \vdots \\ S\left(x_{M},t_{1}\right) & \cdots & S\left(x_{M},t_{N}\right) \end{array}\right)=U_{\mathrm{MxR}}V_{\mathrm{RxN}}$$

The supplied undersampled data **b** allows joint recovery of both U and V according to the optimization

(3C) $$\left\{U*,V*\right\}=\arg\min\left\|F\left(\mathrm{UV}\right)-\right.\;{\left.b\right\|}^{2}+\lambda {\left\|U\right\|}_{l1},\text{such that}\;{\left\|V\right\|}_{F}^{2}<1$$

In addition to the data consistency constraint and the λ‐regularized l_1_‐norm sparsity constraint on the coefficient matrix U, the Frobenius norm of the dictionary V is constrained to assist in convergence ^32^.

In the context of spectroscopic imaging, the basis functions are time‐domain representations of signal from one or more spectral peaks. Working in the time domain rather than the spectral domain yields basis functions that are less orthogonal and helps to promote sparsity in the coefficient matrix U. This approach also avoids biasing the result towards any particular spectral characteristics since the basis functions are learned for each dataset.

To evaluate the impact of undersampling and CS reconstruction, a fully sampled 3D phantom dataset was retrospectively undersampled by a factor of 5.5 with the same CIRCUS sampling strategy used for in vivo mouse data. The results of HSVD‐based fat quantification and saturation indices for each oil sample after Blind‐CS reconstruction were compared with the fully sampled equivalent. The performance of Blind‐CS is controlled primarily by the number R of basis functions in the dictionary and the regularization parameter λ, which controls tradeoff between data consistency and sparsity of the coefficient matrix. For all of the reconstructions presented in this work, the values R = 35 and λ = 0.01 were used; these were empirically determined (data not shown) to minimize the root mean squared error between fully sampled data and data reconstructed with Blind‐CS after undersampling.

### 2.4 Experimental design – phantom

Imaging experiments were performed on a 3 T horizontal bore preclinical system (Oxford Instruments, Abingdon, UK) operated by a Varian/Agilent console, and using a 30 mm i.d. quadrature RF coil (Doty Scientific, Columbia, SC).

A series of phantom experiments was performed to assess the accuracy and stability of quantification with SE‐SPI, as well as its robustness to undersampling and CS reconstruction. NMR tubes (10 mm) were filled with either pure food oils (flax/linseed, peanut, safflower, sesame and soybean) or mixtures of soybean oil and water (20%, 15%, 10% and 5% oil by volume) produced by dilution of Intralipid (Fresenius Kabi, Bad Homburg, Germany). These oils have previously been used to characterize methods for quantifying fatty acid composition ^13^, ^30^, ^33^. Next, 0.16 mM of a gadolinium contrast agent (ProHance, Bracco, Milan, Italy) was added to each oil/water mixture to provide physiologically relevant water T_1_ values.

Tubes were scanned in groups of five using SE‐SPI, using a 64 x 64 matrix for 2D scans or 64 x 64 x 16 for 3D scans, 10 mm slice thickness, 30 x 30 mm field of view (FOV), TR/TE = 200/13 ms, BW = 3 kHz and 512 complex FID points. With full sampling, 2D scans took 13.5 minutes and 3D scans took 3.5 hours. For comparison, a 5 x 5 x 5 mm PRESS spectroscopy voxel was placed over each oil tube and data were acquired using matched TR, TE and BW, with 25 signal averages collected in an acquisition time of ~ 5 seconds. HSVD analysis was performed at each voxel in the SE‐SPI dataset, and for comparison with PRESS, results were averaged across the region matching the PRESS voxel.

### 2.5 Experimental design – animal

To simulate fatty liver disease in an animal model, we supplied BALB/c mice (Charles River, Wilmington, MA) with a methionine and choline deficient (MCD) diet (TD 90262, Teklad Mills, Winfield, IA), which causes large amounts of fat to be sequestered in the liver ^34^, ^35^. Since this process does not lead to iron overload, a common comorbidity in clinical NAFLD ^25^, this was simulated by intravenous injection of superparamagnetic iron oxide (SPIO) particles into the tail vein of a subset of mice 1 day prior to scanning. In total, four groups of four mice each were scanned: one group which received both the MCD diet and iron injections, one which received the MCD diet but no supplemental iron, one which received iron injections but maintained a normal diet, and a final control group with normal diet and no iron injection. The diet, injection and scanning protocol for all animal experiments were approved by the local Research Ethics Board.

Mice receiving the MCD diet were scanned three times: the day before they were started on the MCD diet (day 0), then again after 4 and 11 days. Mice receiving the normal diet were scanned twice, 1 week apart (days 4 and 11). All mice were anesthetized with 3% isofluorane and each had their temperature maintained at 37^0^C (monitored with PC‐Sam, SA Instruments, Stony Brook, NY) using a warm air blower. Anatomical images were obtained using a bSSFP/TrueFISP sequence (256 x 128 x 128 matrix, 0.15 x 0.2 x 0.2 mm voxel size, FA = 30, TR/TE = 8/4 ms), and this was used to plan the placement of the PRESS spectroscopy voxel and the SE‐SPI imaging volume over the liver. 3D SE‐SPI data were acquired using the same parameters as for phantom imaging, with the scan time reduced to 55 minutes using the modified CIRCUS strategy. This represents a prospective undersampling factor of R = 4, which is further increased to ~ R = 5 after respiratory compensation.

**FIGURE 3:**
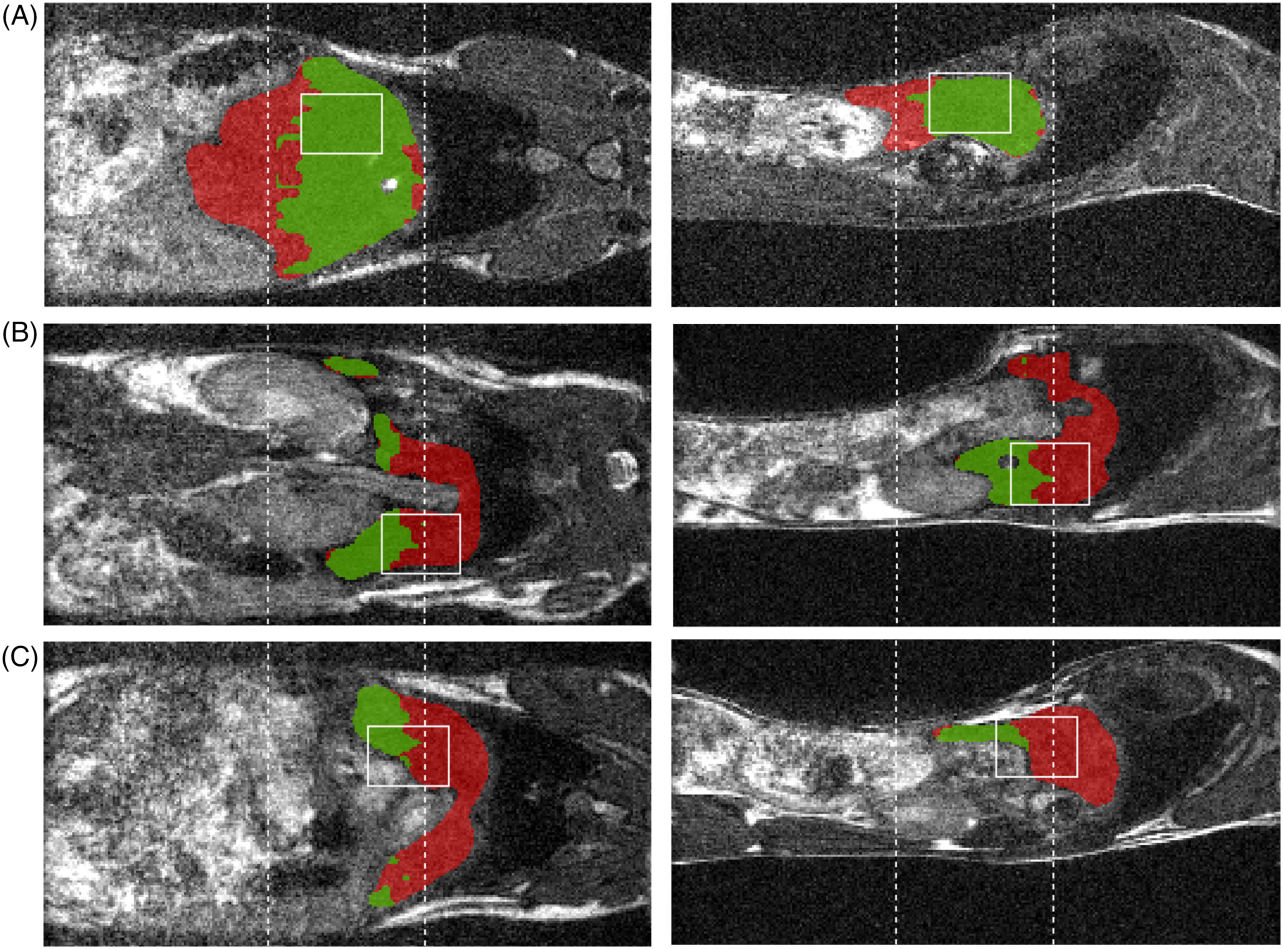
Examples of point resolved spectroscopy (PRESS) voxel placement (solid white box) and spin echo single point imaging (SE‐SPI) field of view (FOV) location (dashed lines) in typical mouse experiments. In all cases the liver (manually segmented in Vivoquant) is shown in red, and liver pixels with acceptable signal intensity in the SE‐SPI acquisition are shown in green. Left column: coronal view; right column: sagittal view. (A) Ideal scenario, in which the PRESS voxel is totally contained within the liver and the SE‐SPI FOV; all green SE‐SPI pixels within the PRESS voxel can be used for comparison. (B) PRESS voxel is entirely within the liver but only some of the useful SE‐SPI pixels overlap with it. Comparisons must involve fewer SE‐SPI pixels or must use some nearby pixels not within the PRESS voxel, assuming local homogeneity. (C) PRESS voxel contains some nonliver tissue, potentially due to movement of the mouse or poor voxel placement, which may reduce the accuracy of fatty acid composition measurements

While efforts were made to ensure that the PRESS and SE‐SPI imaging volumes overlapped (as shown in Figure 3A), in some mice the orientation and position of the liver, or effects of respiratory motion, made this challenging (ie, Figure 3B,C). To make the best use of the available data in these instances, a region of interest (ROI) encompassing the entire liver was drawn in VivoQuant (Invicro, Boston, MA) based on the bSSFP anatomical image. The SE‐SPI data were aligned with the anatomical, and those voxels within the drawn ROI which had suitable signal intensity (more than the mean signal intensity in the image) were used for the comparison with PRESS. This criterion was empirically selected to yield ROIs with consistently high SNR.

Of the 36 mouse datasets collected, 23 had a PRESS voxel with at least 50% of its volume overlapping the high‐intensity SE‐SPI volume, and in most of the remaining cases (eg, Figure 3B), the PRESS voxel did overlap with liver tissue contiguous with the SE‐SPI ROI. The overall percentage of overlap between PRESS voxels and SE‐SPI ROIs was 54 +/‐ 32% (mean +/‐ standard deviation). One mouse in the MCD diet group on day 0 of the study had an SE‐SPI ROI which contained almost no voxels of suitable intensity, and the PRESS voxel overlapped with none of them; this data point was excluded from further analysis.

One mouse from each group was sacrificed following the day 4 scan, and all mice were sacrificed after the day 11 scan. Livers were removed from the mice, suspended in a fluorinated hydrocarbon solution and scanned again with PRESS (using a voxel large enough to cover the entire excised liver, usually 15 x 15 x 15 mm) to provide an ex vivo measurement of fat concentration for comparison with the in vivo results. After computation of fat fraction and saturation indices from all three scans (in vivo PRESS, ex vivo PRESS and in vivo SE‐SPI), values were compared between groups using a Welch test (a paired t‐test which does not assume equal variances). A significance level of α = 0.05 was used, with a Bonferroni correction for multiple comparisons applied as appropriate; eg, with n = 6 comparisons for each scan, the *P*‐value needed for significance was 0.05/6 = 0.0083.

## 3 RESULTS

**FIGURE 4:**
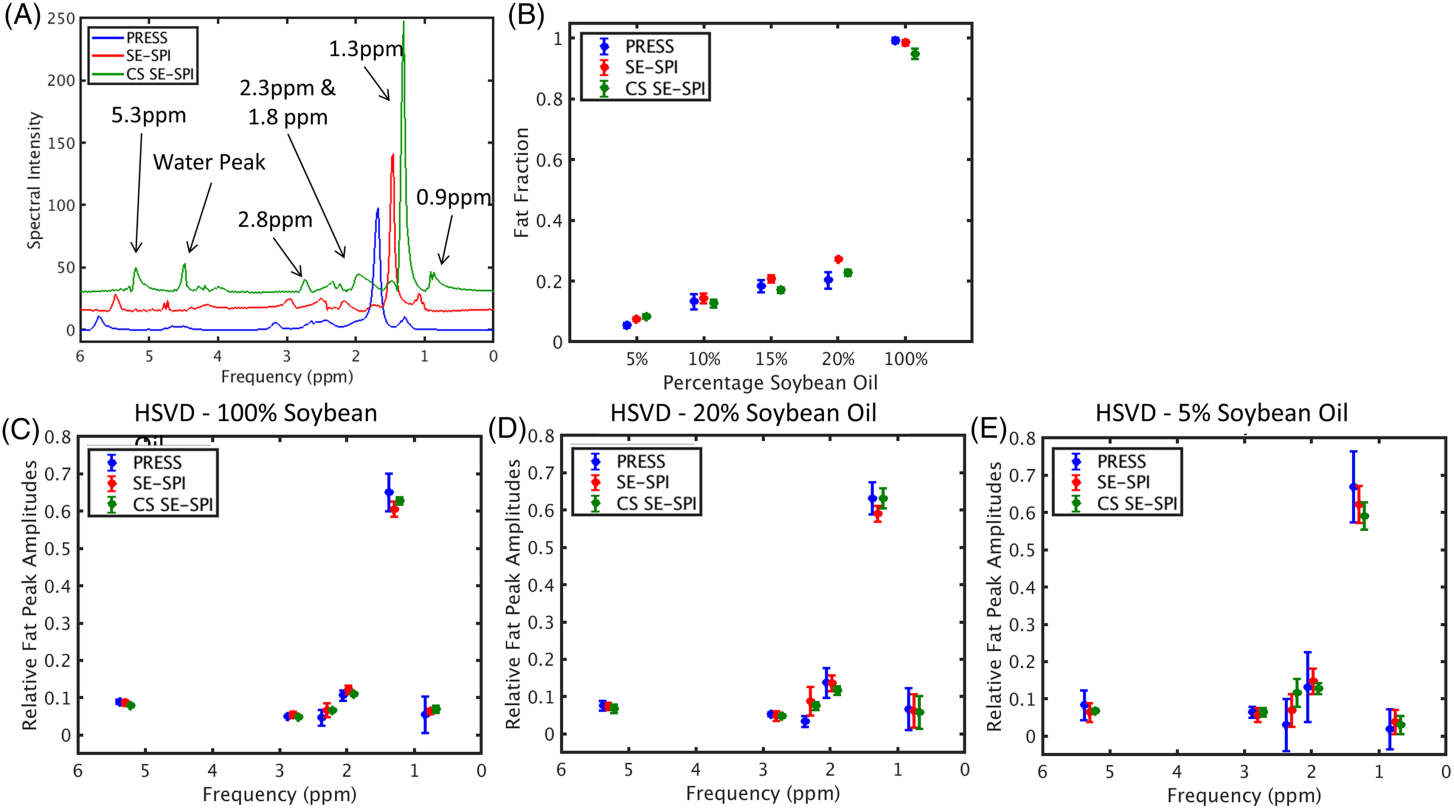
Comparison of point resolved spectroscopy (PRESS) and spin echo single point imaging (SE‐SPI) for fat quantification in mixed oil/water phantoms. (A) PRESS, SE‐SPI and compressed sensing (CS) SE‐SPI spectra (unphased, displaying absolute value) showing peaks of a 100% soybean oil sample, offset for clarity. Note the presence of a small water peak after CS reconstruction due to aliased signal from nearby tubes. (B) Fat fractions computed by Hankel singular value decomposition (HSVD)‐based spectral analysis, with measurements from different sequences shifted slightly for ease of visualization. (C) Relative fat peak amplitudes of a 100% soybean oil tube, performed using data from PRESS (blue), fully sampled SE‐SPI (red) and SE‐SPI after retrospective undersampling at R = 5.5 and CS reconstruction (green). For visualization, PRESS and CS results are offset from SE‐SPI by ‐0.08 and 0.08 ppm, respectively. (D) Relative fat peak amplitudes of a 20% soybean oil/80% water phantom. (E) Relative fat peak amplitudes of a 5% soybean oil phantom. Points and error bars in all plots represent mean and 95% confidence interval based on five independent measurements

Figure 4 shows a comparison of PRESS and SE‐SPI fat quantification in mixtures of soybean oil and water ranging from 5% to 100% oil. The PRESS spectrum of Figure 4A shows the six peaks used for HSVD analysis. Fat fraction and composition measurements are consistent across all tubes, even after undersampling and reconstruction with Blind‐CS (green markers in Figure 4B‐E), with larger variability at lower fat fractions as anticipated. The most significant differences in fat fraction estimation between PRESS and SE‐SPI after CS reconstruction are at 5% and 100% fat fraction. As shown in Figure 4A, in cases where the fat content differs significantly from the average of the entire image, residual aliased signal from nearby tubes may be amplified by the Blind‐CS reconstruction, which has a small though visible impact on the estimated fat fraction.

**FIGURE 5:**
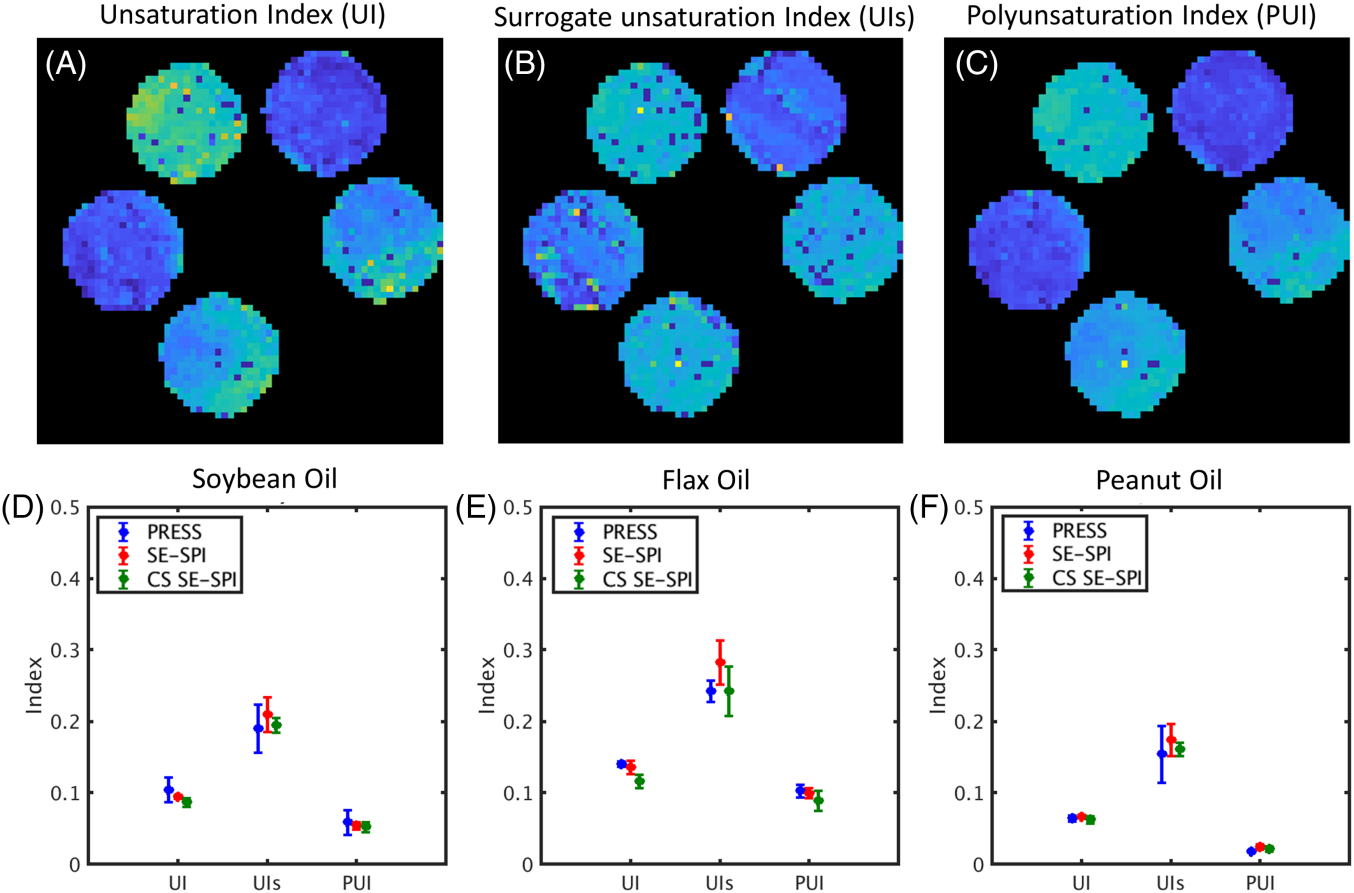
Demonstration of Hankel singular value decomposition (HSVD)‐based fat composition profiling in pure oil phantoms. (A) Example unsaturation index (UI) map based on compressed sensing (CS) spin echo single point imaging (SE‐SPI) data from five pure oils (clockwise from top left: flax, peanut, sesame, soybean and safflower). (B) Example surrogate unsaturation index (UIs) map. (C) Example polyunsaturation index (PUI) map. (D) Comparison of saturation indices for soybean oil, computed based on data from point resolved spectroscopy (PRESS) (blue), fully sampled SE‐SPI (red) and SE‐SPI after retrospective undersampling at R = 5.5 and CS reconstruction (green). SE‐SPI results are shifted with respect to PRESS for ease of visualization. (E) Saturation indices for flax oil. (F) Saturation indices for peanut oil. Points and error bars in all plots represent mean and 95% confidence interval based on five independent measurements

Figure 5 demonstrates spatially resolved mapping of unsaturation indices in pure oils using SE‐SPI after CS reconstruction. The three derived indices (UI, UIs and PUI) are generally homogeneous throughout individual tubes, and show significant differences between different oil types, which are known in the literature to have different degrees of saturation ^29^. While the HSVD decomposition did fail in some individual pixels, in the majority of voxels the undersampling and reconstruction process does not have a significant impact upon measurements of saturation indices in pure oils. Figure S1 shows that these indices are stable with respect to changes in fat fraction in phantoms of the same oil type.

**FIGURE 6:**
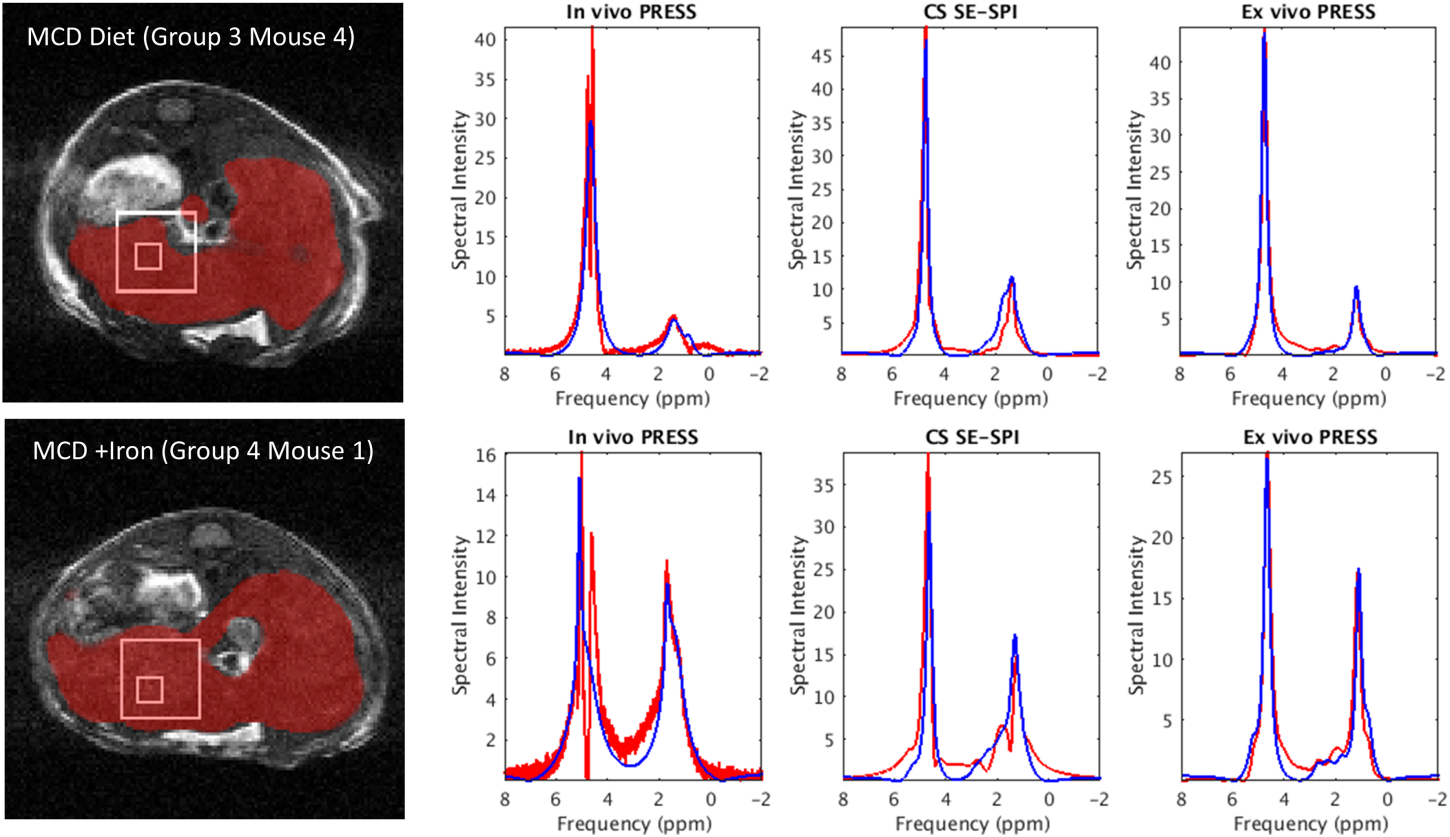
Typical spectra from mice receiving the methionine and choline deficient (MCD) diet. Top row: a mouse receiving the MCD diet but no supplemental iron injections. The leftmost panel shows the TrueFISP anatomical image with the liver region of interest (ROI) overlain in red. The large white box indicates the approximate location of the point resolved spectroscopy (PRESS) voxel; the small white box indicates the location of a 3 x 3 x 3 voxel sub‐ROI of spin echo single point imaging (SE‐SPI) data used for the accompanying plot. Spectra shown are, from left to right, in vivo PRESS (red = raw, blue = Hankel singular value decomposition [HSVD] fit), in vivo SE‐SPI (red = raw data averaged over 3 x 3 voxel ROI, blue = HSVD fit to 3 x 3 x 3 voxel ROI), ex vivo PRESS. Bottom row: the same plots but for a mouse receiving the MCD diet and supplemental iron injections; note the increased line width and reduced SNR of the in vivo PRESS voxel, but little change in SE‐SPI

Typical spectra from PRESS and SE‐SPI are shown in Figure 6. Both mice in this example received the MCD diet, and received either no supplemental iron injections (top row) or received them before each scan (bottom row). In the first case, some of the lower amplitude fat peaks necessary for computing the saturation indices are visible in both PRESS and SE‐SPI spectra, although the SE‐SPI spectrum exhibits narrower line widths and better resolution of peaks, most likely due to its smaller effective voxel size. For the mouse which received iron injections, the line widths are all visibly broadened, and only SE‐SPI retains some of the lower amplitude peaks (eg, between 2 and 3 ppm, and 5.3 ppm).

**FIGURE 7:**
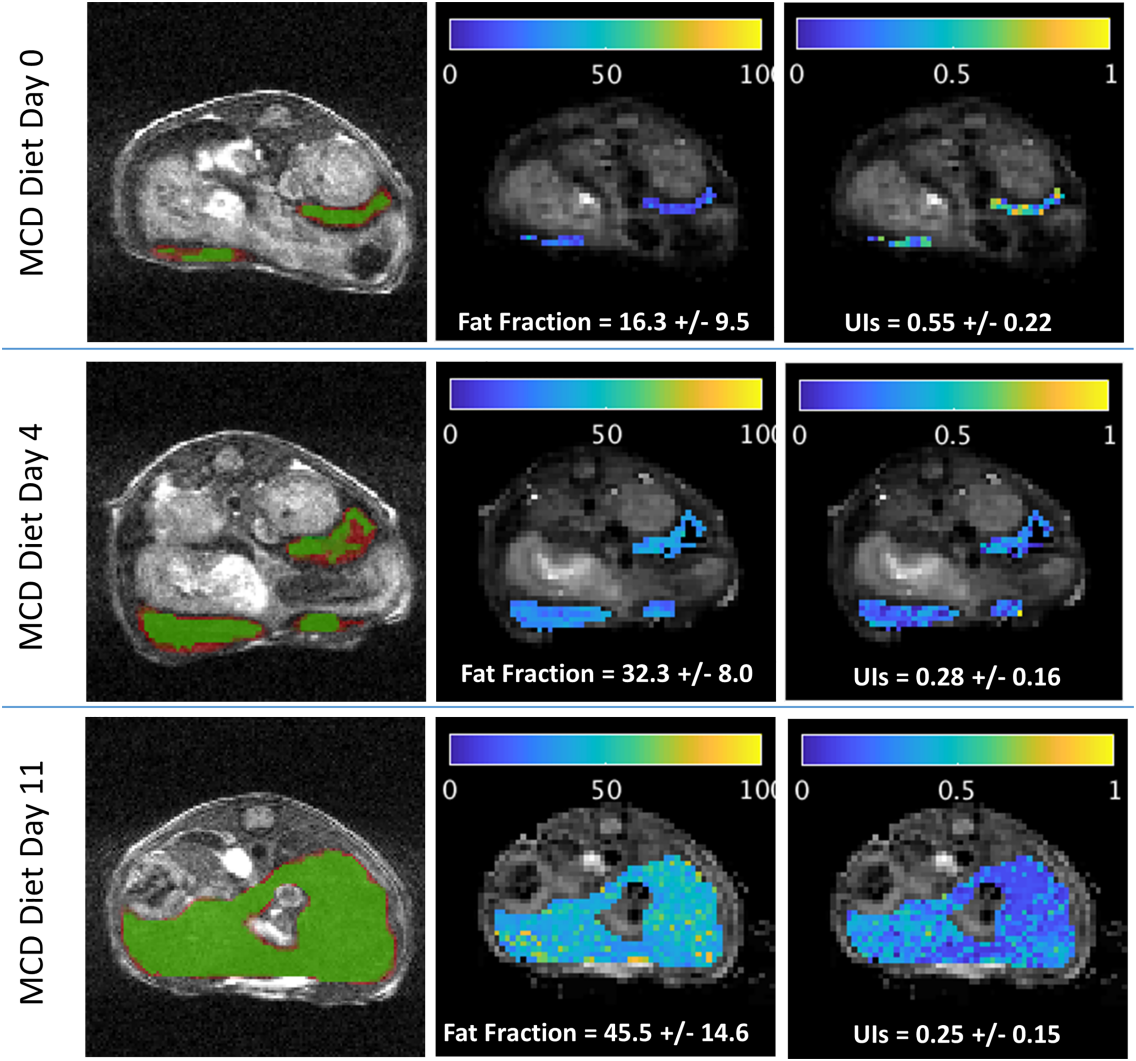
Example of spin echo single point imaging (SE‐SPI)‐based quantification of fat content and composition in a mouse receiving the methionine and choline deficient (MCD) diet and supplemental iron injections (Group 4, Mouse #1), as measured on Day 0 (top row), Day 4 (middle row) and Day 11 (bottom row) of the diet. The leftmost panel in each row shows the TrueFISP anatomical, with the liver region of interest (ROI) overlain in red, and the SE‐SPI ROI in green (voxels in red ROI with high SE‐SPI signal and with edges eroded). Slices displayed are through the center of the SE‐SPI volume and may not reflect the entire liver extent. The central and rightmost panels show maps of the fat fraction and surrogate unsaturation index (UIs) calculated at each voxel of the SE‐SPI ROI, overlain on the SE‐SPI image (after compressed sensing [CS] reconstruction). Reported values are mean +/‐ standard deviation over the ROI

Figure 7 illustrates the progression of fat accumulation in the liver of an individual mouse scanned on days 0, 4 and 11 of the MCD diet, as demonstrated by spatially resolved maps of fat fraction computed based on the HSVD at each voxel. The rightmost panels also show the UIs maps for the mouse, which may indicate changes in composition happening over this timescale. These results are from one of the mice that received supplemental iron, but are typical of the entire MCD cohort.

**FIGURE 8:**
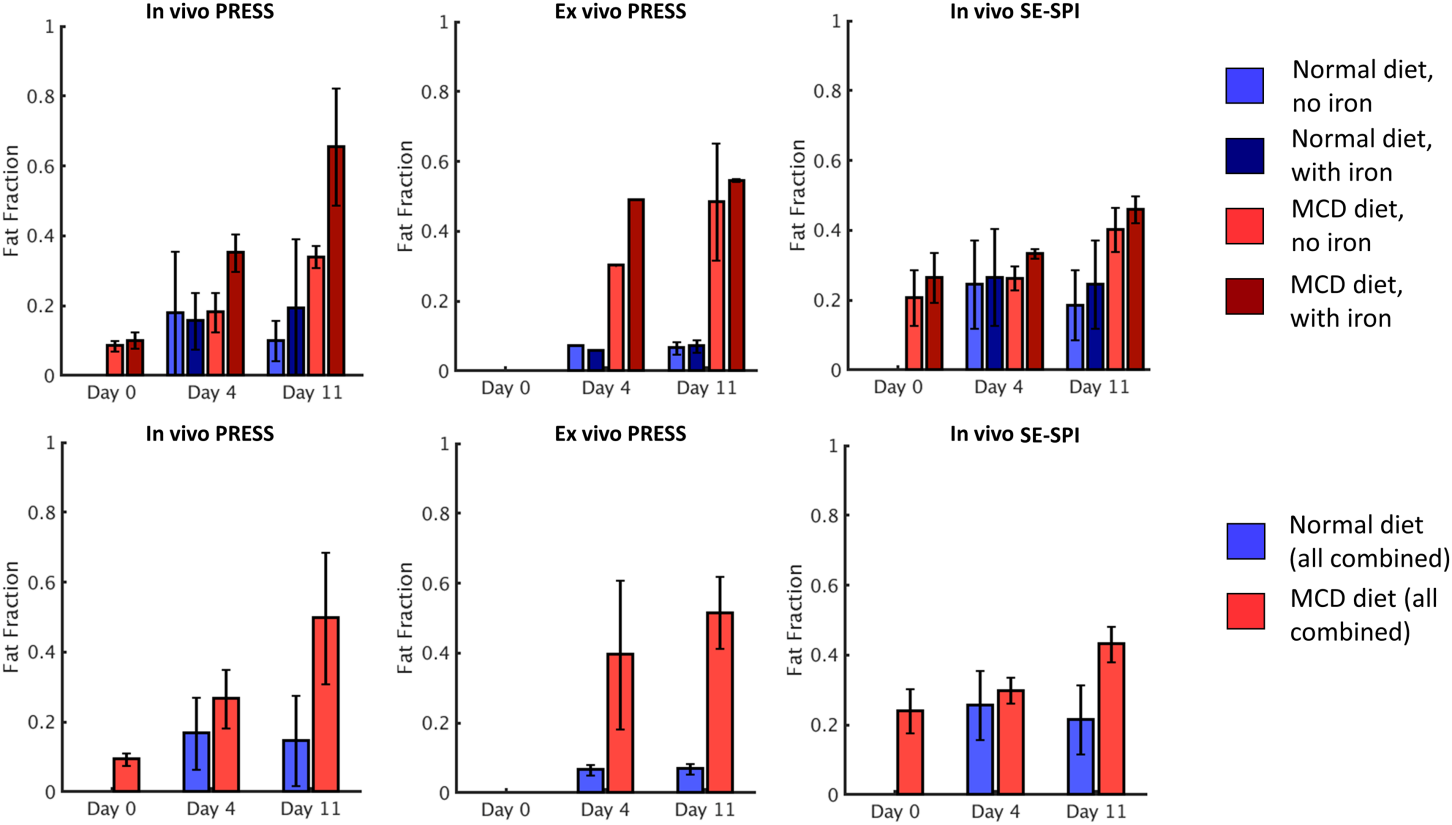
Fat fraction of all mouse livers as measured by point resolved spectroscopy (PRESS) (in vivo and ex vivo) and compressed sensing (CS) spin echo single point imaging (SE‐SPI). Data in the top row are grouped into the four experimental groups; bar height is the average fat fraction across the group and error bars represent the 95% confidence interval. Data in the bottom row are grouped by diet only, ie, mice which did and did not receive iron injections but did receive the same diet are considered together. Note that no ex vivo scans were performed on day 0, and the mice receiving the normal diet were scanned only on Day 4 and Day 11

Figure 8 summarizes the measurement of fat fraction using PRESS (in vivo and ex vivo) and SE‐SPI at the group level. (Figure S2 shows the breakdown of data at the individual level, although a direct point‐by‐point comparison is not always possible due to nonoverlapping fields of view; see eg, Figure 3). The overall trend for fat fraction measurement is similar in both PRESS and SE‐SPI, both when separated into four experimental groups (top row) and when the data from all mice receiving the same diet are considered together (bottom row). A significant increase is seen in all the MCD‐receiving mice, as was expected given the fat sequestration induced by this diet, while the mice receiving normal diets showed no significant overall trend across two scans. When the groups are considered separately, in vivo PRESS measurements appear to overestimate the fat fraction in MCD diet mice that received iron compared with MCD diet mice that received no iron, whereas SE‐SPI does not show a significant difference, as was expected since injected iron should have no impact upon fat content.

In general, the quality of the PRESS data was generally not sufficient to reliably compute saturation indices for many of the mice (see Table 1), whereas most SE‐SPI data yielded consistently detectable peaks and indices, even in those mice that had received iron injections. PUI was most challenging to measure with SE‐SPI, but on average 40% of all SE‐SPI liver voxels yielded a nonzero value of this metric.

**TABLE 1:** Fraction of spectra with detected peaks suitable for Hankel singular value decomposition (HSVD) analysis. For spin echo single point imaging (SE‐SPI), values refer to the average number of liver region of interest (ROI) voxels in which a nonzero value of the stated index was calculated (ie, the peak[s] in the numerator of the index were detected and had nonzero amplitude). For point resolved spectroscopy (PRESS), values refer to the number of acquired liver voxels in which a nonzero index value was calculated

Modality	Mouse group	Viable UI	Viable UIs	Viable PUI
SE‐SPI	Group 1 (normal diet/no iron)	56 +/‐ 21%	97 +/‐ 3%	38 +/‐ 23%
	Group 2 (normal diet/iron)	58 +/‐ 17%	98 +/‐ 2%	43 +/‐ 27
	Group 3 (MCD diet/no iron)	70 +/‐ 21%	95 +/‐ 5%	46 +/‐ 32%
	Group 4 (MCD diet/iron)	60 +/‐ 22%	93 +/‐ 8%	33 +/‐ 21
	Overall	62 +/‐ 23%	95 +/‐ 6%	40 +/‐ 26%
In vivo PRESS	Group 1 (normal diet/no iron)	3/7	4/7	2/7
	Group 2 (normal diet/iron)	5/7	5/7	4/7
	Group 3 (MCD diet/no iron)	7/11	5/11	2/11
	Group 4 (MCD diet/iron)	8/11	7/11	5/11
	Overall	23/36 (64%)	21/36 (58%)	13/36 (36%)
Ex vivo PRESS	Group 1 (normal diet/no iron)	4/4	3/4	3/4
	Group 2 (normal diet/iron)	1/4	1/4	1/4
	Group 3 (MCD diet/no iron)	4/4	4/4	4/4
	Group 4 (MCD diet/iron)	4/4	4/4	4/4

Abbreviations: MCD, methionine and choline deficient; PUI, polyunsaturation index; UI, unsaturation index; UIs, surrogate unsaturation index.

At the group level, neither PRESS nor SE‐SPI showed significant changes in the UI or PUI over the course of the MCD diet. (PUI, as measured by SE‐SPI, showed a decreasing trend when data from all mice receiving the same diet were combined, but the trend was not statistically significant.) The breakdown of individual data for these indices is shown in Figures S3 and S4, with plots summarizing the group level results in Figures S6 and S7.

**FIGURE 9:**
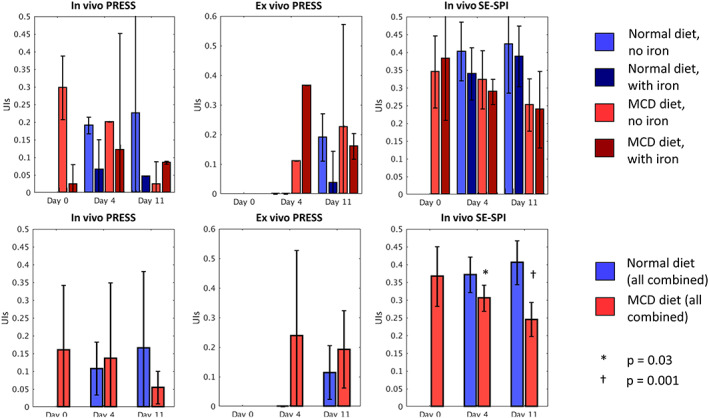
Surrogate unsaturation index (UIs) of all mouse livers as measured by point resolved spectroscopy (PRESS) (in vivo and ex vivo) and compressed sensing (CS) spin echo single point imaging (SE‐SPI). Data in the top row are grouped into the four experimental groups; bar height is the average unsaturation index across the group and error bars represent the 95% confidence interval. Data in the bottom row are grouped by diet only. Measurements showing statistically significant differences between methionine and choline deficient (MCD) and normal diet groups are indicated; only the difference on Day 11 remains significant after correction for multiple comparisons

One significant difference was observed in the UIs values at the group level, which are summarized in Figure 9 (individual results in Figure S5). When all mice receiving the same diet are considered together, SE‐SPI measurements of UIs showed a significant decrease at day 11 in the MCD group compared with the normal diet group; the decrease at day 4 was significant before Bonferroni correction but not afterwards. When split into four groups, the trend remains, although the significance is reduced due to the smaller sample size. This may indicate that the UIs is sensitive to changes in fatty acid composition during the initial stages of accumulation in the liver, which is consistent with the literature examining the impact of this diet formulation in mice ^34^.

## 4 DISCUSSION

Proper characterization of NAFLD is essential to patient management, and the limitations to traditional biopsy‐based screening are increasingly being addressed by noninvasive biomarkers such as MRI‐based measurements of PDFF. While methods to measure PDFF have achieved significant clinical deployment, PDFF is not a strong predictor of eventual disease progression, despite its strong correlation with liver steatosis ^10^, ^11^. Biomarkers such as the indices proposed in ^13^ or ^31^ may be more relevant for such purposes, and techniques to compute them accurately over the entire liver could be a valuable diagnostic aid for patients known to have early‐stage NAFLD.

The MCD diet used in this mouse model may not fully reproduce the changes in fatty acid composition seen in NAFLD, since NAFLD involves different biological mechanisms that occur over significantly different timescales. Nonetheless, the ability of SE‐SPI to discern changes in saturation indices related to the MCD diet indicates that such techniques may be useful in determining whether similar changes are observed in patient populations.

It should be noted that the saturation indices used in this work are not identical with the actual chemical proportion of saturated fat in the liver, nor is the reported fat fraction necessarily identical to the volume fraction of fatty acids. This is due to T_1_ saturation caused by short repetition times, a limitation inherent to many practical fat quantification techniques. Differences between the T_1_ of water and fat mean that spectral peak areas are not a direct measure of fat concentration without appropriate compensation, and similarly, differences in T_1_ between individual peaks within the fat spectrum mean that relative amplitudes of those peaks are also dependent upon the TR of the sequence.

However, the ultimate application of this work is not absolute quantification of fat spectra, but reliable identification of spectral features that may be useful in studying particular disease models over time. Metrics such as the UIs, which reflect the relative importance of certain fat peaks, still have potential use as a diagnostic biomarker if they can be reproducibly measured, which should be possible with SE‐SPI at a fixed TR and field strength. Indeed, the T_1_ dependence of the saturation indices may be an asset; if further studies show particular peaks in the fat spectrum to be of interest in monitoring liver disease, then knowledge of the T_1_ of those peaks may inform a choice of scan parameters that maximizes their contribution to the overall signal.

Reliably identifying the spectral components needed for calculation of the saturation indices can be challenging, and due to insufficient signal or other artifacts, the HSVD analysis was not always able to discriminate all of the six desired spectral peaks in each supplied signal. When this occurs (eg, if the 2.8 ppm diallylic methylene peak is not found), the corresponding saturation indices are also set to zero, and are omitted from the analysis. Table 1 summarizes the number of in vivo and ex vivo PRESS voxels, and the proportion of SE‐SPI voxels, which yielded nonzero values of the three indices computed. It can be seen that a significant fraction of the in vivo PRESS spectra are not suitable for computation of the saturation indices, likely due to a combination of respiratory motion and inhomogeneous B_0_ over the larger voxel. Even ex vivo PRESS (which covers the entire excised liver, is unaffected by respiratory motion and should have more uniform B_0_) fails to capture all of the fat peaks in the normal diet group. Future experiments exploring the limits of quantification accuracy with SE‐SPI must strive to overcome these factors through optimized PRESS voxel size and placement, increased signal averaging or more advanced respiratory compensation strategies.

In the present work, however, rather than using either PRESS measurement as a gold standard against which the saturation values obtained with SE‐SPI can be validated, we sought to determine whether SE‐SPI could reliably measure changes in saturation indices that are consistent with how this particular diet should impact fat composition. The literature generally indicates that the MCD diet should decrease the proportion of polyunsaturated fat. Although no significant change in UI or PUI was observed (reflecting the difficulty in resolving the 5.3 and 2.8 ppm peaks even with SE‐SPI), there was a decreasing trend in PUI (see Figure S7) and UIs did show a significant decrease after 11 days, consistent with predictions from the literature. As indicated by Table 1, enough voxels have nonzero UIs to lend confidence to these measurements.

In clinical applications, fat measurements made with traditional single‐voxel MR spectroscopy are more reliable than the preclinical data presented here; the use of larger voxels and multichannel RF arrays to boost SNR mean that the small peaks necessary to compute saturation metrics can be more easily resolved. More advanced motion compensation techniques are also available. Nonetheless, SVS remains limited by spatial coverage and can fall prey to some of the same drawbacks as biopsy in that it can mischaracterize heterogeneous tissues, including the liver, which is known to exhibit nonuniform fat accumulation in NAFLD ^9^, and may be further exacerbated by changes in cholesterol levels (ie, fatty acid composition) ^36^. Spectroscopic imaging methods such as SE‐SPI that can cover entire slices or volumes of liver tissue provide a more complete picture of liver composition. These methods also generally enable smaller voxel sizes, improving field homogeneity and reducing line‐broadening effects, eg, as evidenced in Figure 6. This is of particular importance in livers with iron overload, a common comorbidity of fatty liver disease. Our results show that measurements of the saturation indices can still be achieved in the presence of significant liver iron.

The major obstacle to clinical deployment of MRSI techniques is acquisition time; without some form of acceleration, even a modest 32 x 32 x 8 matrix would require almost 30 minutes of scan time at a minimal TR of 200 ms. Compensation for respiratory motion is also a necessity for any abdominal applications. Our approach to both these problems was to use a variable density sampling strategy based on CIRCUS, which oversamples the center to ensure sufficient coverage after respiratory compensation, while undersampling the periphery to reduce the overall scan time. For the in vivo mouse scans in this work we used a high‐resolution 3D sampling trajectory (64 x 64 x 16), which cannot be acquired on a clinical system in a reasonable scan time, even with multichannel RF arrays to provide further acceleration via parallel imaging. However, high‐resolution 2D (64 x 64) and lower resolution 3D (40 x 40 x 8) patterns are both possible, and we are now exploring which is most appropriate for clinical validation.

The use of undersampling and CS always carries the potential for loss of information, usually in the form of high‐resolution, low‐contrast features which do not fit assumptions of sparsity in the model‐based reconstruction. The algorithm used in this work, Blind‐CS, attempts to discern the underlying temporal basis functions from a time‐resolved dataset from the data itself, assuming that a small number of such basis functions can adequately describe the signal evolution. It is possible that, in a situation where fatty acid composition differs significantly throughout an imaging volume, the algorithm will assign greater weight to the more dominant component and suppress others. The stability of saturation indices in the pure oil phantoms (Figure 5) before and after CS reconstruction implies that, if present, this effect is not overwhelming, but further investigation of the robustness of Blind‐CS in this context is warranted and will be the subject of future work. This could potentially be done in silico to allow testing of a wider range of conditions than would be encountered in vivo.

## 5 CONCLUSIONS

In this work we have demonstrated that free‐breathing, accelerated spectroscopic imaging can provide spatially resolved measurements of fatty acid composition, both in oil phantoms and in an in vivo animal model of fatty liver disease. SE‐SPI generally agrees with SVS measurements, but is able to provide reasonable estimates of saturation indices based on individual peak heights in many cases where SVS cannot adequately resolve the necessary spectral features due to line broadening. Clinical testing of this technique can now be undertaken to assess whether saturation indices can provide a reliable imaging biomarker for severity or progression of NAFLD.

## FUNDING INFORMATION

This work was supported by an Investigator Sponsored Research Agreement with GE Healthcare, funding from the Atlantic Canada Opportunities Agency's Atlantic Innovation Fund, a Brain Canada Technology and Platform Grant, a National Sciences and Engineering Research Council Discovery Grant (S.D.B.), a Canadian Institutes of Health Research Canada Graduate Scholarship (M.H.) and a Killam Predoctoral Scholarship (M.H.).

## Supporting information

Supporting Information S1

Supporting Information S2

Supporting Information S3

Supporting Information S4

Supporting Information S5

Supporting Information S6

Supporting Information S7
