# Supplementary figures and images for: Mapping of fatty acid composition with free‐breathing MR spectroscopic imaging and compressed sensing
Source: NMR Biomed. 2020 Jan 3;34(5):e4241. doi: 10.1002/nbm.4241 (PMC8244113; doi:10.1002/nbm.4241)

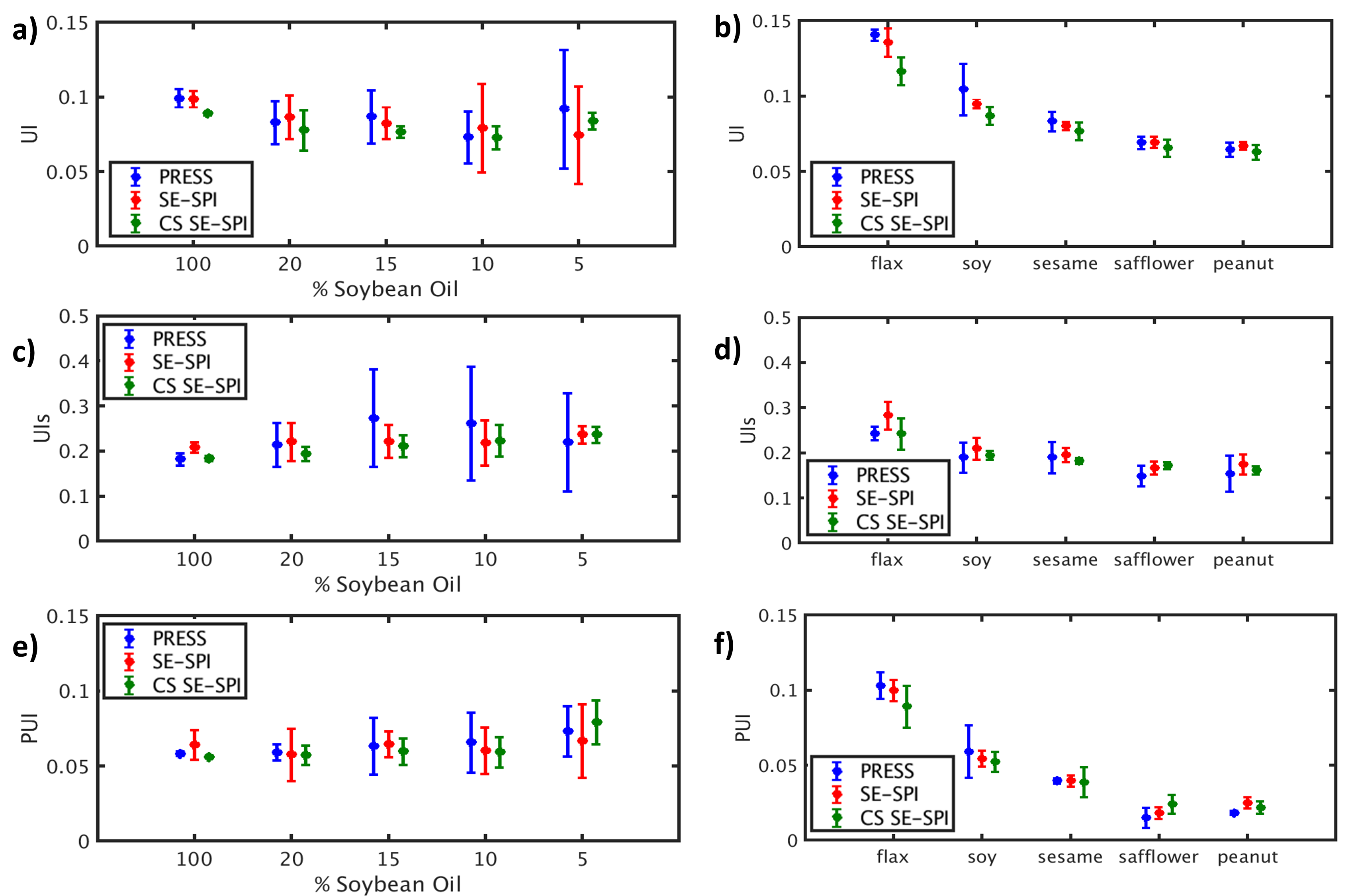

Supplement: Supplementary file 1 — Supporting Information S1 [file NBM-34-e4241-s005.tif]

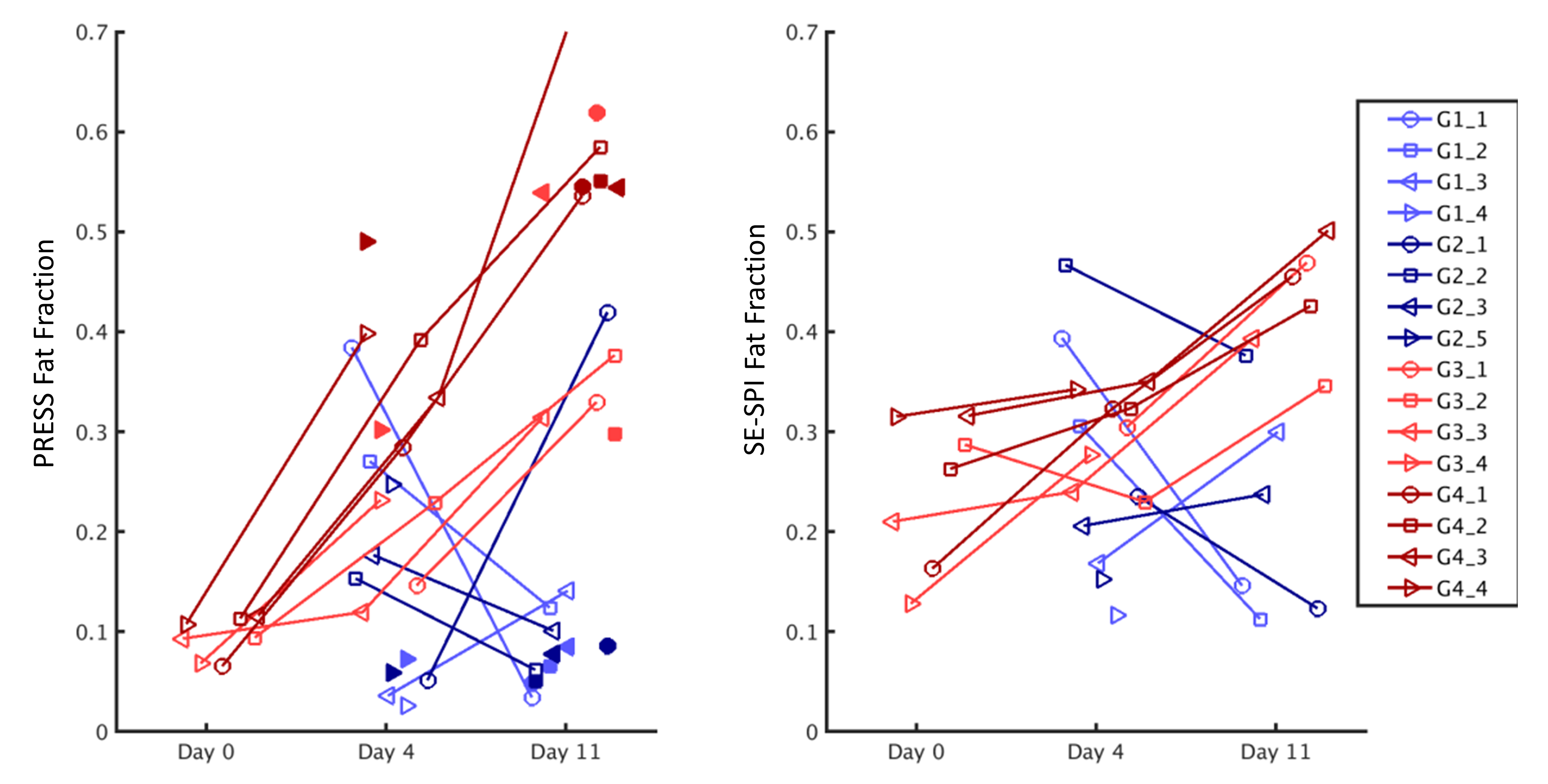

Supplement: Supplementary file 2 — Supporting Information S2 [file NBM-34-e4241-s004.tif]

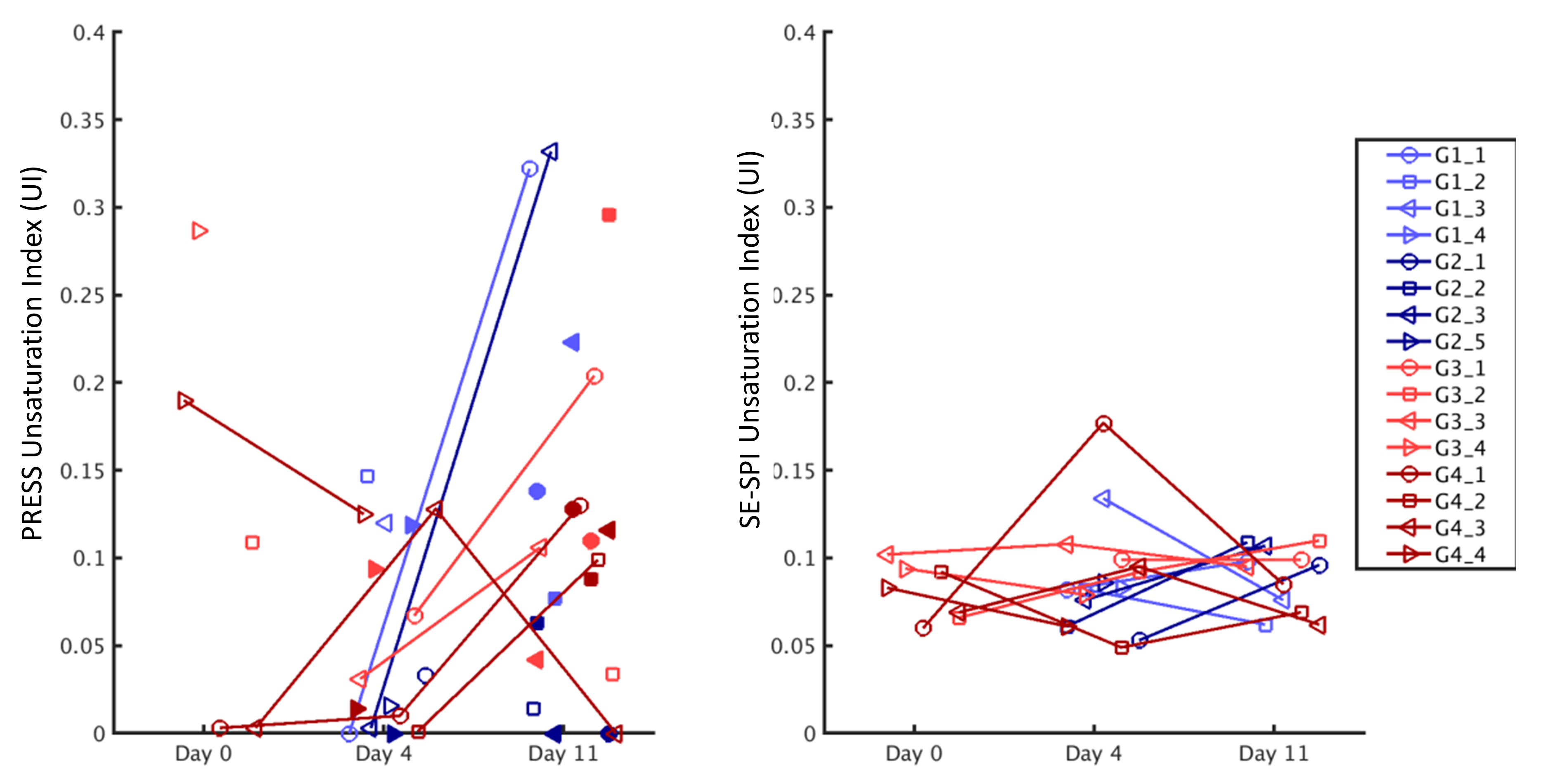

Supplement: Supplementary file 3 — Supporting Information S3 [file NBM-34-e4241-s003.tif]

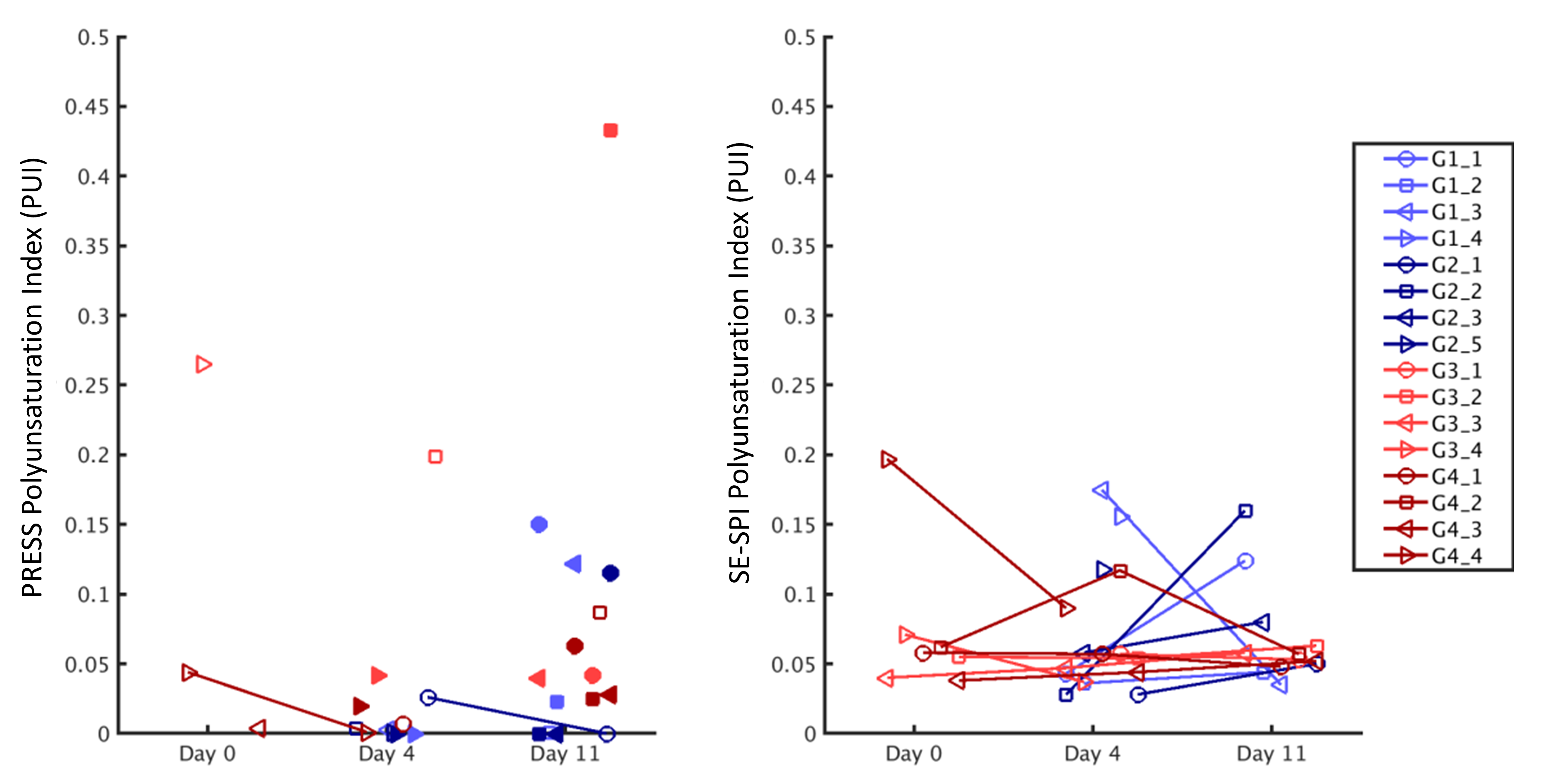

Supplement: Supplementary file 4 — Supporting Information S4 [file NBM-34-e4241-s007.tif]

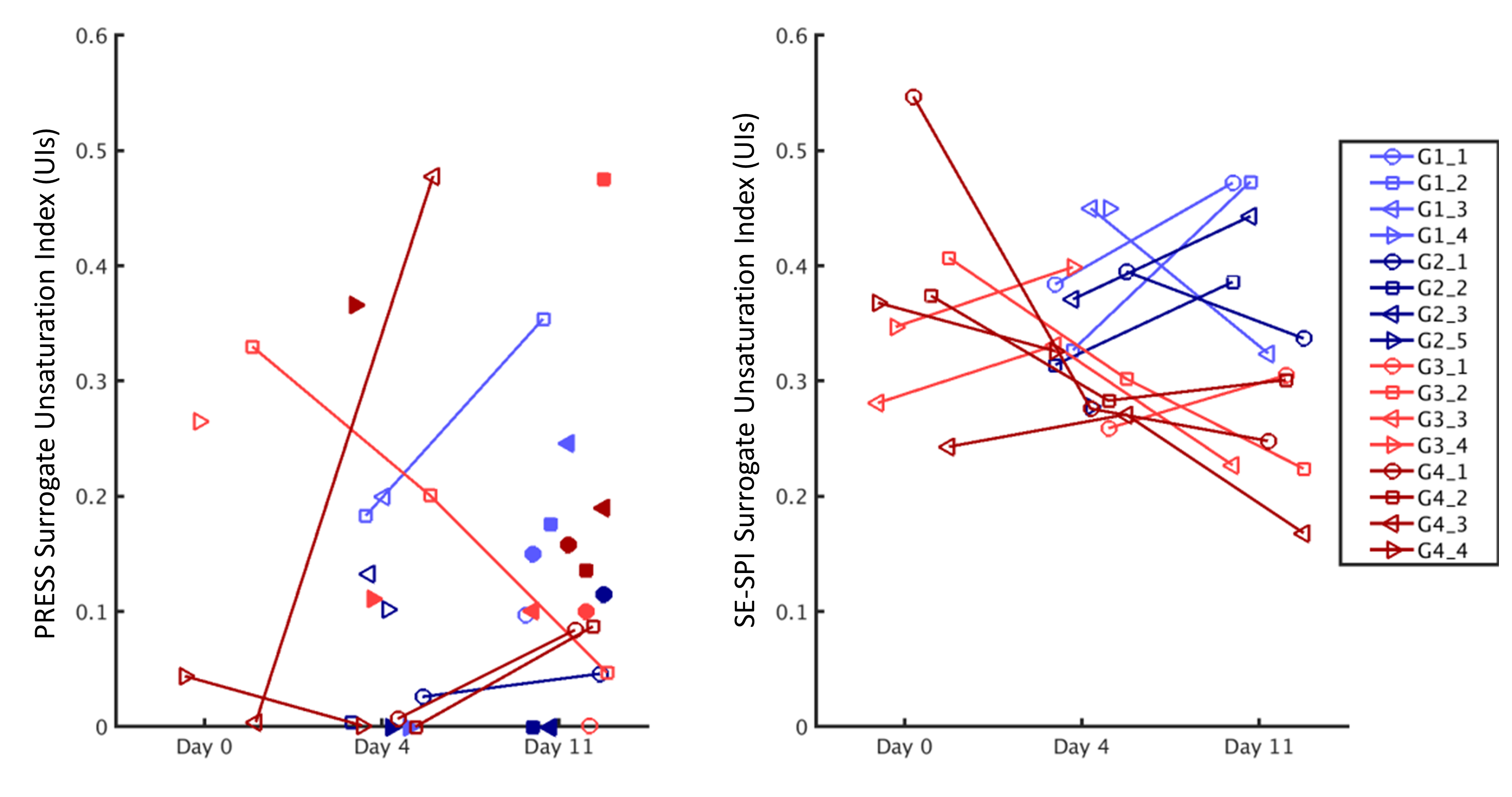

Supplement: Supplementary file 5 — Supporting Information S5 [file NBM-34-e4241-s006.tif]

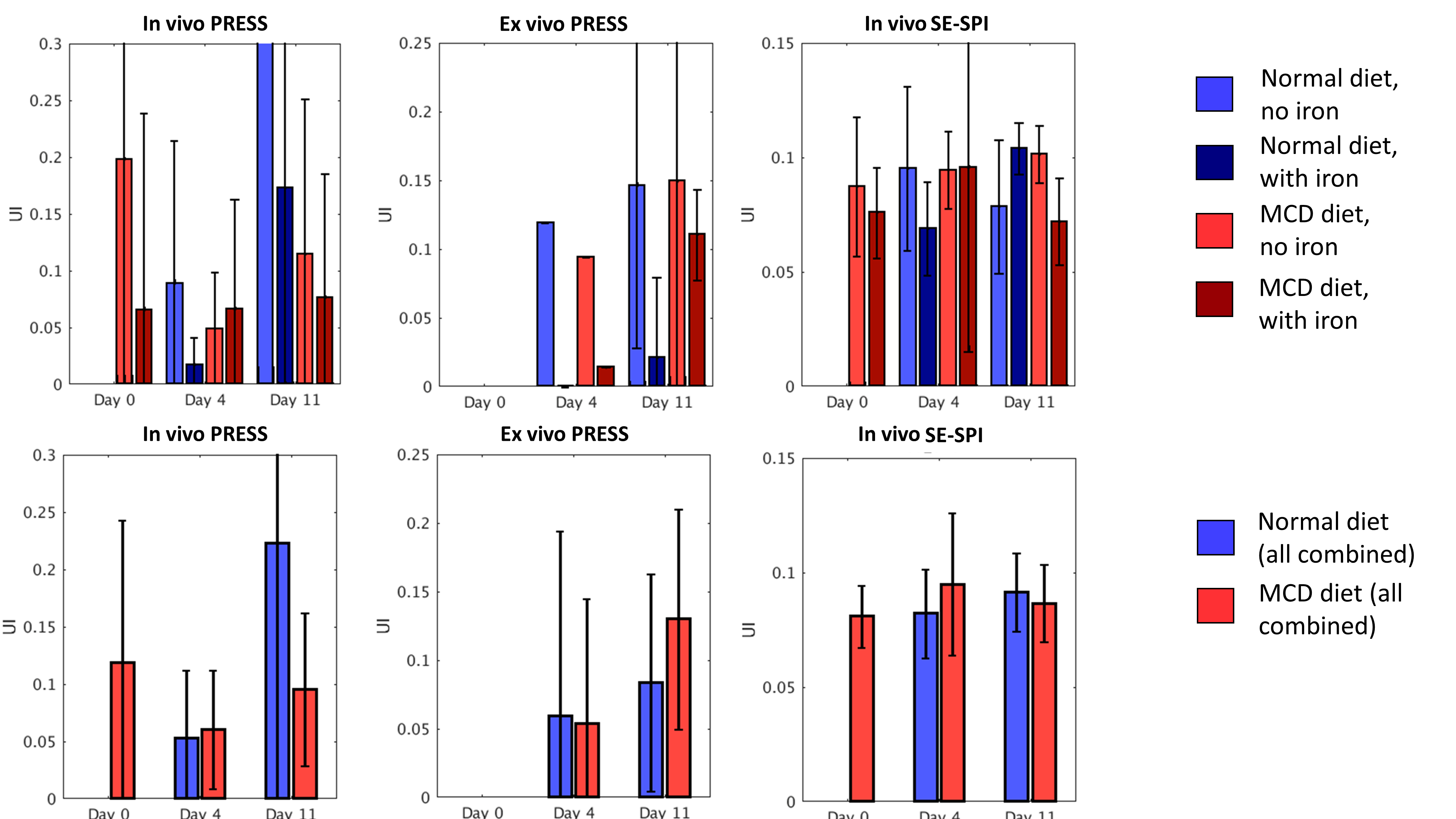

Supplement: Supplementary file 6 — Supporting Information S6 [file NBM-34-e4241-s002.tif]

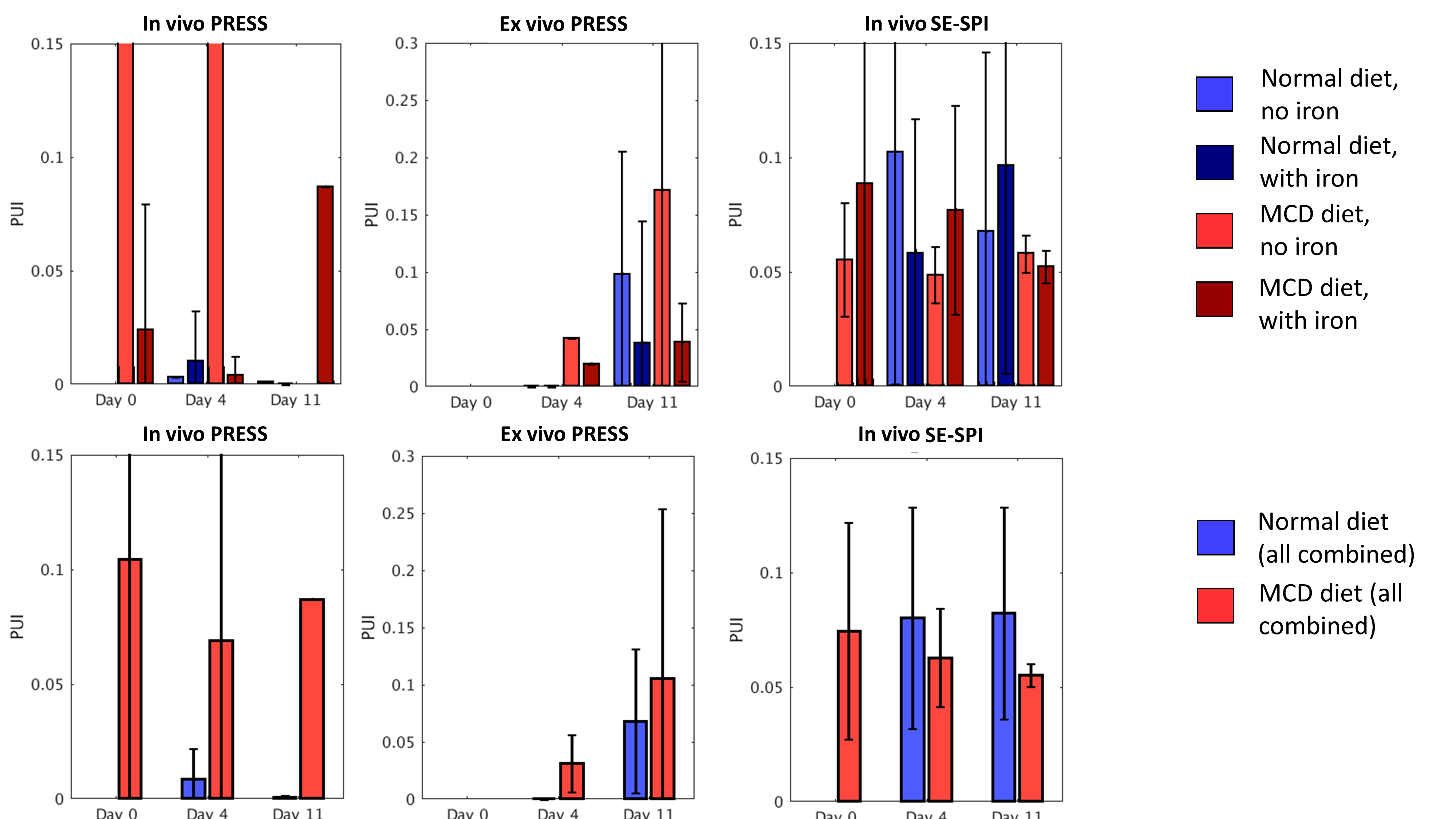

Supplement: Supplementary file 7 — Supporting Information S7 [file NBM-34-e4241-s001.tif]
